# Physiotherapy Post Lumbar Discectomy: Prospective Feasibility and Pilot Randomised Controlled Trial

**DOI:** 10.1371/journal.pone.0142013

**Published:** 2015-11-12

**Authors:** Alison Rushton, Nicola R. Heneghan, Melanie Calvert, Alison Heap, Louise White, Peter C. Goodwin

**Affiliations:** 1 School of Sport, Exercise and Rehabilitation Sciences, College of Life and Environmental Sciences, University of Birmingham, Birmingham, United Kingdom; 2 Primary Care Clinical Sciences, School of Health and Population Sciences, College of Medical and Dental Sciences, University of Birmingham, Birmingham, United Kingdom; 3 Physiotherapy Department, University Hospitals Birmingham NHS Foundation Trust, Queen Elizabeth Hospital, Birmingham, United Kingdom; 4 Health Professions Department (Physiotherapy), Manchester Metropolitan University, Birley Fields, Manchester, United Kingdom; Copenhagen University Hospital, Hvidovre, DENMARK

## Abstract

**Objectives:**

To evaluate: acceptability and feasibility of trial procedures; distribution of scores on the Roland Morris Disability Questionnaire (RMDQ, planned primary outcome); and efficient working of trial components.

**Design and Setting:**

A feasibility and external pilot randomised controlled trial (ISRCTN33808269, assigned 10/12/2012) was conducted across 2 UK secondary care outpatient physiotherapy departments associated with regional spinal surgery centres.

**Participants:**

Consecutive consenting patients aged >18 years; post primary, single level, lumbar discectomy.

**Interventions:**

Participants were randomised to either 1:1 physiotherapy outpatient management including patient leaflet, or patient leaflet alone.

**Main Outcome Measures:**

Blinded assessments were made at 4 weeks post surgery (baseline) and 12 weeks post baseline (proposed primary end point). Secondary outcomes included: Global Perceived Effect, back/leg pain, straight leg raise, return to work/function, quality of life, fear avoidance, range of movement, medication, re-operation.

**Results:**

At discharge, 110 (44%) eligible patients gave consent to be contacted. 59 (54%) patients were recruited. Loss to follow up was 39% at 12 weeks, with one site contributing 83% losses. Mean (SD) RMDQ was 10.07 (5.58) leaflet and 10.52 (5.94) physiotherapy/leaflet at baseline; and 5.37 (4.91) leaflet and 5.53 (4.49) physiotherapy/leaflet at 12 weeks. 5.1% zero scores at 12 weeks illustrated no floor effect. Sensitivity to change was assessed at 12 weeks with mean (SD) change -4.53 (6.41), 95%CI -7.61 to -1.44 for leaflet; and -6.18 (5.59), 95%CI -9.01 to -3.30 for physiotherapy/leaflet. RMDQ mean difference (95%CI) between change from baseline to twelve weeks was 1.65(-2.46 to 5.75). Mean difference (95%CI) between groups at 12 weeks was -0.16 (-3.36 to 3.04). Participant adherence with treatment was good. No adverse events were reported.

**Conclusions:**

Both interventions were acceptable, and it is promising that they both demonstrated a trend in reducing disability in this population. A randomised controlled trial, using a different trial design, is needed to ascertain the effectiveness of combining the interventions into a stepped care intervention and comparing to a no intervention arm. Findings will guide design changes for an adequately powered randomised controlled trial, using RMDQ as the primary outcome.

**Trial Registration:**

ISRCTN registry 33808269

## Introduction

Low back pain (LBP) affects 80% of the population within their lifetime [1] and contributes to lost productivity and sickness/disability benefit estimated at £10,668million annually [2] and resulting in 3.51 million lost working days.[3] Inpatient surgical treatment is the largest single component of expenditure in managing LBP.[2] Lumbar discectomy surgery is conducted to excise part of a prolapsed intervertebral disc for a primary indication of leg pain. It is estimated that 1.2 billion euros annually are required to cover the direct and indirect costs of patients experiencing leg pain.[4] Lumbar discectomy operations in the UK National Health Service (NHS) were performed for 8478 patients (mean age 45 years) in the 2013/2014 year.[5] The mean hospital stay for first-time lumbar discectomy of 2.3 days (2013/2014) equates to 16,685 National Health Service bed days.[5] Data from the Netherlands estimates 12,000 operations per year [6] and from the United States 287,122 operations.[7] Although the success rate of lumbar discectomy is reported as 46–75% at 6–8 weeks, and 78–95% at 1–2 years post surgery,[8] post-operative rehabilitation is a key issue, with 30% to 70% of patients experiencing residual pain,[9] 3% to 12% seeking further surgery,[10] and only 70% fit to return to work 12 months after surgery;[11] especially when considering the low mean age for this procedure. In the UK in 2013/2014 1164 (approximately 14%) revision operations were performed.[5]

A UK audit of spinal surgeons[12] identified that post-operative advice was variable. For example, the period recommended for 'no sitting' ranged from 2 to 42 days. Routine referral of patients for physiotherapy after discharge was made by 55% of surgeons. A survey of physiotherapy management post lumbar discectomy[13] identified that individual, out-patient physiotherapy was provided routinely in 44% of UK spinal centres and in a further 46% of centres in situations where patients had residual problems. The content and advice provided were very variable e.g. number of sessions ranged from 1 to 20, and a wide range in type of exercises prescribed. The surveys highlighted the need for research to optimise rehabilitation for this patient group. 49% centres had access to classes / group sessions.[13]

Our recent systematic review[14] evaluated effectiveness of physiotherapy intervention post first single level lumbar discectomy at 3 months (short term) and 6 months (longer term), on clinically relevant outcomes of disability, function and health. All physiotherapy outpatient interventions were included. The majority of trials involved group rehabilitation. Three trials investigated individualised (1:1) physiotherapy management, which is reflective of current practice[13] in several countries including the UK. The review identified only one of 16 trials as having low risk of bias. Some evidence supported that physiotherapy improved disability in the short-term, with a potential benefit of a more intensive intervention. Weak evidence supported improved movement and physical impairment, in the short-term. Overall, there was inconclusive evidence for effectiveness of outpatient physiotherapy post first lumbar discectomy. Group rehabilitation was subsequently evaluated in the UK by McGregor et al[15] who found no benefit of ‘your back operation’ booklet (not surgery specific) or group rehabilitation intervention following discectomy.

An updated Cochrane systematic review of rehabilitation programmes post lumbar disc surgery[8] included 22 trials, with 10 assessed as low risk of bias. Key findings included: programmes commencing immediately after surgery were no more effective than control; low/very low quality evidence supporting no differences between rehabilitation programmes commencing 4–6 weeks after surgery; low quality evidence supporting physiotherapy commencing at 4–6 weeks compared to no treatment/education only; and that multidisciplinary rehabilitation led by medical advisors led to faster return to work than usual care. Statistical pooling was limited but illustrated a potential positive effect of exercise on pain and function; with very low/low quality evidence supporting high intensity exercise programmes as more effective than low-intensity in the short term. Interestingly, very low quality evidence suggested no significant differences between supervised and home exercise programmes to achieve this.

Both systematic reviews identified great variability of the content and duration of interventions and outcomes and that no moderate or high quality evidence is available. Both reviews called for a low risk of bias adequately powered trial, with evidence supporting a physiotherapy intervention, inclusive of intensive exercise commencing at 4 weeks post surgery. However the nature of the intervention and the trial design required careful consideration. A feasibility and external pilot trial was therefore conducted to inform a multi-centre randomised controlled trial (RCT) comparing effectiveness and cost effectiveness of two interventions post first lumbar discectomy. The objectives were to evaluate acceptability and feasibility of trial procedures, evaluate the distribution of scores on the Roland Morris Disability Questionnaire (RMDQ), and evaluate if the components of the trial work efficiently together.

### Specific objectives

1. To evaluate acceptability and feasibility of individual procedures, including:
  - Recruitment strategy[16,17]
  - Eligibility criteria[16]
  - Randomisation to the two interventions[16,17]
  - Blinding procedures[16,17]
  - Data collection (including use of data collection forms)[16]
  - Follow up procedures at 4 weeks post surgery (baseline) and, 12 and 26 weeks post baseline[16,17]
2. To evaluate acceptability of interventions
3. To appraise the best way of providing information to patients to enable them to make an informed decision about participating in the RCT.
4. To determine whether different procedures work together[17]
5. To evaluate:
  - Consent rate[17,18]
  - The training for the different roles that physiotherapists undertook in the trial
  - Participant adherence rates[19]
6. To assess feasibility of acquiring the required sample size in a realistic time-scale[19]
7. To evaluate the distribution of scores on the RMDQ for use in the targeted population to:
  - Inform its appropriateness, in particular with regards to potential floor effects[19]
  - Enable estimation of the standard deviation of scores to inform the sample size calculation for an adequately powered RCT[19]

## Materials and Methods

### Trial design

A feasibility and external pilot trial was conducted according to a pre-defined protocol (S1 Protocol). This was a small scale parallel 1:1 RCT design. Consenting patients across two sites were randomised to either individualised 1:1 physiotherapy outpatient management including patient leaflet (physiotherapy/leaflet), or patient leaflet alone. Blinded assessments were made at 4 weeks post surgery (baseline) and 12 weeks post baseline (proposed primary end point RCT). 50% participants were followed up at 26 weeks.

### Participants

#### Inclusion criteria

Male and female patients aged >18 years; post primary, single level, lumbar discectomy (including microdiscectomy),[20] able to communicate in English.

#### Exclusion criteria

Previous surgery at the same spinal level; co-morbidities that might impact on ability to participate in trial interventions including cauda equina, cognitive dysfunction, uncontrolled cardiovascular disease,[20] osteoporotic fracture, spondylolythesis, multiple sclerosis, tumour; complications from surgery such as excessive bleeding, severe intra-operative root damage, level error, or severe wound infection[20,21] that would prevent participation in either intervention; and participation in a concurrent trial.

Patients were invited to participate prior to discharge following surgery. Patients who were interested in participating were given a copy of the Participant Information Sheet. An *Introducer* (physiotherapist working on the surgical ward) discussed the trial with patients and answered any questions, checked eligibility if interested in participating, and requested written consent from eligible patients to be contacted to arrange an out-patient appointment 4 weeks post surgery. The *Introducer* gave the patient a Patient Leaflet and discussed it, answering any questions.

At 4 weeks post surgery, a *recruiter* (physiotherapist working in outpatient department) discussed the Patient Leaflet again and answered any questions. They assessed eligibility of patients willing to participate and obtained formal, written consent. *Recruiters* randomised participants and advised the participant whether they would need to attend hospital for an assessment at 12 weeks or at both 12 and 26 weeks. With their consent, participants’ GPs were notified of their participation. Separate written consent was obtained for participation in the focus groups at the beginning of each group, following explanation of the purpose of the focus group and what participation would entail.

The setting was 2 outpatient physiotherapy departments in the UK associated with regional spinal surgery centres—the Queen Elizabeth Hospital Birmingham (QEHB) and Salford Royal NHS Foundation Trust (SRFT). Patients recruited at QEHB lived 2 to 31 miles from the department, and patients at SRFT lived 3 to 35 miles away; the possible large distances reflecting the hospitals being regional centres.

### Interventions

#### Physiotherapy/leaflet

The 1–1 physiotherapy intervention encompassed education, advice, mobility exercises, core stability exercises, a progressive approach to exercise to increase intensity, and encouragement of early return to work and activity; with patients attending up to 8 physiotherapy sessions, over a period of up to 8 weeks (to allow for patient choice and variations in practice at each trial site), starting at 4 weeks post surgery to provide optimal care.[8,14] It incorporated flexibility for physiotherapists to tailor management to individual patients, thereby ensuring patient centred practice, in line with MRC guidance for developing and evaluating complex interventions.[22] The intervention was designed to reflect best practice, based on best evidence.[8,13,14,23] It was developed and agreed by the research team, clinical experts and spinal surgeons at 5 spinal centres (planned sites for the RCT), physiotherapists and patients; and is detailed in full elsewhere.[24] Physiotherapists delivering the intervention included n = 8 introducers initially delivering the leaflet component (bands 5–7) and n = 4 (experienced bands 5 and 6) treating physiotherapists delivering the 1:1 component at the SRFT site, and n = 4 introducers (bands 6–8) and n = 3 (band 7) treating physiotherapists at the QEHB site.

#### Patient leaflet

No surgery-specific leaflet existed nationally; therefore, the Patient Leaflet was developed through a 3-round Delphi study, from information in existing leaflets at the 5 spinal centres and focus groups involving patients and clinicians.[25] The Delphi study used a purposive sample (n = 51) of experts including spinal surgeons, inpatient and outpatient physiotherapists and patients post lumbar discectomy from the 5 spinal centres. The Patient Leaflet included sections on: anatomy, disc herniation, surgery, activity post surgery, exercises and their progression, and frequently asked questions.

### Outcomes

Outcome assessment was 4 weeks after surgery (baseline), and following intervention at 12 weeks post baseline. Follow-up assessment at 26 weeks post baseline was planned for 50% of participants in each intervention group to assess the feasibility of longer term follow up. Assessments were recorded on a case report form and comprised both patient reported and performance based outcome measures. Demographic data including age, gender, duration of symptoms prior to surgery, planned or emergency surgery, presence of leg and/or back pain, whether taking analgesia, employment status, ethnicity, and distance from centre were collected to describe participant characteristics. All patient reported outcomes were completed by participants at baseline and follow-up(s) appointments with support from the blinded assessor as required. All performance based outcomes were evaluated by the blinded assessor.

The primary outcome for the RCT was planned as the RMDQ, an extensively used disease-specific measurement tool for low back pain, with established properties of reliability and validity.[26] Evidence indicated good discrimination for patients with mild to moderate disability and pre-eminence for use post-lumbar disc surgery.[27] Minimum clinically important change is reported as 3.5 points.[28] The RMDQ is a 24 item scale, scored 0–24 with 0 indicating no dysfunction, and completion takes 5 minutes.

The choice of secondary measures was informed by patients, surgeons, and physiotherapists to ensure their importance as no core set of outcomes exists (www.comet-initiative.org). The primary aim was not to overburden participants, whilst collecting data that covered all important outcomes post lumbar discectomy i.e. body function, body structures, activities and participation, and environmental factors in accordance with the International Classification of Functioning, Disability and Health.[29] Each measure was used in at least one trial included in our systematic review.[14]

- **Global Perceived Effect (GPE)** is a self-report measure of a patient’s perceived effect of treatment,[28,30] rated 1 to 7, where 1 = completely recovered, 2 = much improved, 3 = slightly improved, 4 = not changed, 5 = slightly worse, 6 = much worse and 7 = worse than ever compared with pre-surgery.
- **Visual Analogue Scale (VAS) leg pain and VAS back pain** is measured 0-10cm, with 0 “no pain” and 10 “worst pain ever”.[28] Both were reported for “today”, “least level of pain over the past 2 weeks”, and “greatest level of pain over the past 2 weeks”. VAS is responsive in a chronic LBP population[31] with a reported MCID of 2.0.
- **EQ-5D 5L** was used to measure health-related quality of life, and will inform a cost utility analysis in the future RCT.[31] Data regarding postcode, employment, use of healthcare resources and associated costs was also collected to test feasibility of data collection for the future cost utility analysis.
- **Time to return to work / normal activities /full duty** (as relevant) was recorded in days from date of operation. It is a key outcome for LBP research particularly when evaluating prognosis.[32]
- **Tampa Scale for Kinesiophobia (TSK)** is a 17 item scale with each item rated as 1 “strongly disagree”, 2 “disagree”, 3 “agree”, or, 4 “strongly agree”; that provides a measure of pain related fear. It has established reliability and validity in patients with LBP.[33]
- **Fear Avoidance and Beliefs Questionnaire (FABQ**) is a self-report measure of fear avoidance. Some evidence supports its reliability, validity and responsiveness albeit in translated versions.[34] Although there is some overlap between the TSK and FABQ both were used to evaluate their application to this post-surgical population where fear avoidance was anticipated.
- **Straight Leg Raise (SLR)** was measured in cm and normalised for leg length. It is a sensitive test in patients with signs and symptoms of nerve root involvement.[35]
- **Range of lumbar movement**: The modified Schober method was used to measure range of flexion, extension and side flexion (left and right) in the lumbar spine. The method has established properties of excellent reliability and moderate validity.[36]
- **Use of analgesia** was recorded as leg pain is the main indication for surgery.
- **Revision operation** as suggested to be a factor illustrative of poor outcome.[5,10]

In addition, at their final assessment (either 12 or 26 weeks post baseline), every participant was asked to indicate their level of adherence with their exercises recommended in the Patient Leaflet, and, for participants in the physiotherapy/leaflet group, their level of adherence with any additional home exercises recommended by the physiotherapist. Their use of physiotherapy or other intervention (e.g. chiropractic) outside the trial was also recorded, as an indication of potential dilution of treatment effect if taken up by participants in the Patient Leaflet only group; or, as an indication of potential inflation effect if taken up by patients in the 1:1 group. In addition, qualitative data from focus group interviews were collected, and will be reported in a separate qualitative paper with a clear reference to the primary trial.

### Sample size

No formal sample size calculation was performed, as the objectives related to recruitment, retention and the feasibility and acceptability of the trial. Investigations of changes in key trial parameters were exploratory. Thirty patients[37,38] were required in each intervention arm at the 12 week assessment (primary end point RCT) to provide sufficient insight into recruitment and retention rates, and to allow estimates of variability and change scores to be calculated for the RMDQ.

### Randomisation

#### Sequence generation

Consenting patients at the two sites were randomised to one of the two interventions using telephone randomisation accessing a random allocation sequence. Allocation had equal weighting to the two interventions, and 50% follow-up at 26 weeks per group. There was one deviation from the trial protocol as a decision was made not to stratify based on scores of RMDQ (<15 and ≥15).

#### Allocation concealment mechanism

Participants and *recruiters* were blind to treatment allocation prior to the point of allocation. No one apart from the telephone contact had access to allocation codes.

#### Implementation

The random allocation sequence was generated by the trial statistician. The *recruiter* obtained the allocated intervention by telephone at the 4 week appointment while the baseline assessment was conducted. Following the baseline assessment, the *recruiter* assigned the participant to the intervention and then had no further participant contact.

### Blinding

The nature of the two interventions prohibited blinding participants and treating physiotherapists to the allocated treatment, a well-recognised limitation to controlling potential sources of bias when evaluating complex interventions.[39,40] In order to minimise bias *recruiters* asked each participant not to discuss any treatment with *assessors* (physiotherapists undertaking blinded outcome assessment). *Assessors* were masked to allocation and did not take part in recruitment, allocation or treatment processes; collecting data in an area of the physiotherapy department separate to the treatment area. To evaluate blinding, at both 12 and 26 weeks, *assessors* were asked to indicate which intervention they thought participants had received.

### Data analysis

A CONSORT diagram[41] was used to describe the flow of participants through the trial. This information summarised the feasible eligibility, recruitment and follow-up rates. Statistical analyses were performed using IBM SPSS, version 21. Analysis was focused to descriptive statistics. Data were summarised as n (%), mean (Standard Deviation[SD]) or median (interquartile range), as appropriate, to characterise the overall sample and each group. Participants who received a treatment other than that randomised remained in the trial and their data were included in intention to treat analyses.

The distribution of scores on the RMDQ were considered at baseline and at 12 and 26 weeks follow-up. The % of zero scores at 12 weeks was used as the measure of a potential floor effect. Evidence of a large floor effect would cast serious doubt on the choice of RMDQ as the primary, disease-specific, measurement tool in the RCT. If there were no evidence of a large floor effect, baseline data and change scores of RMDQ at 12 weeks were used to estimate the standard deviation (and 95% confidence intervals) for scores and change scores, respectively. These values would support the calculation of sample size for the RCT. In addition, estimated values for the mean and standard deviation would be compared with values published in similar trials.[8,14]

### Research governance

The trial was conducted in accordance with the Research Governance Framework for Health and Social Care, in the context of its feasibility and pilot nature. R&D approval was gained from both sites. The West Midlands–Solihull Research Ethics Committee granted ethical approval (Ref: 12/WM/0224, 25^th^ September 2012). A Study Management Group involving users monitored trial progress and addressed any management, ethical or academic issues. A Study Steering Group involving users (combined Trial Steering Group and Data Monitoring & Ethics Committee) reviewed relevant information from the trial team to oversee trial progress towards achieving its objectives; considered any adverse events; and protected the rights of trial participants. All data were collected using case report forms (including patient intervention data), and anonymised data were stored securely at the University.

Procedures for reporting and serious adverse events were established. An intervention would have been withdrawn if it led to an unacceptable number of serious or adverse events for individuals randomised to it. A serious adverse event was defined as one that required hospitalisation as a result of the intervention, or where treatment caused unwarranted distress to a participant.

## Results

### Participant flow

**Fig 1:**
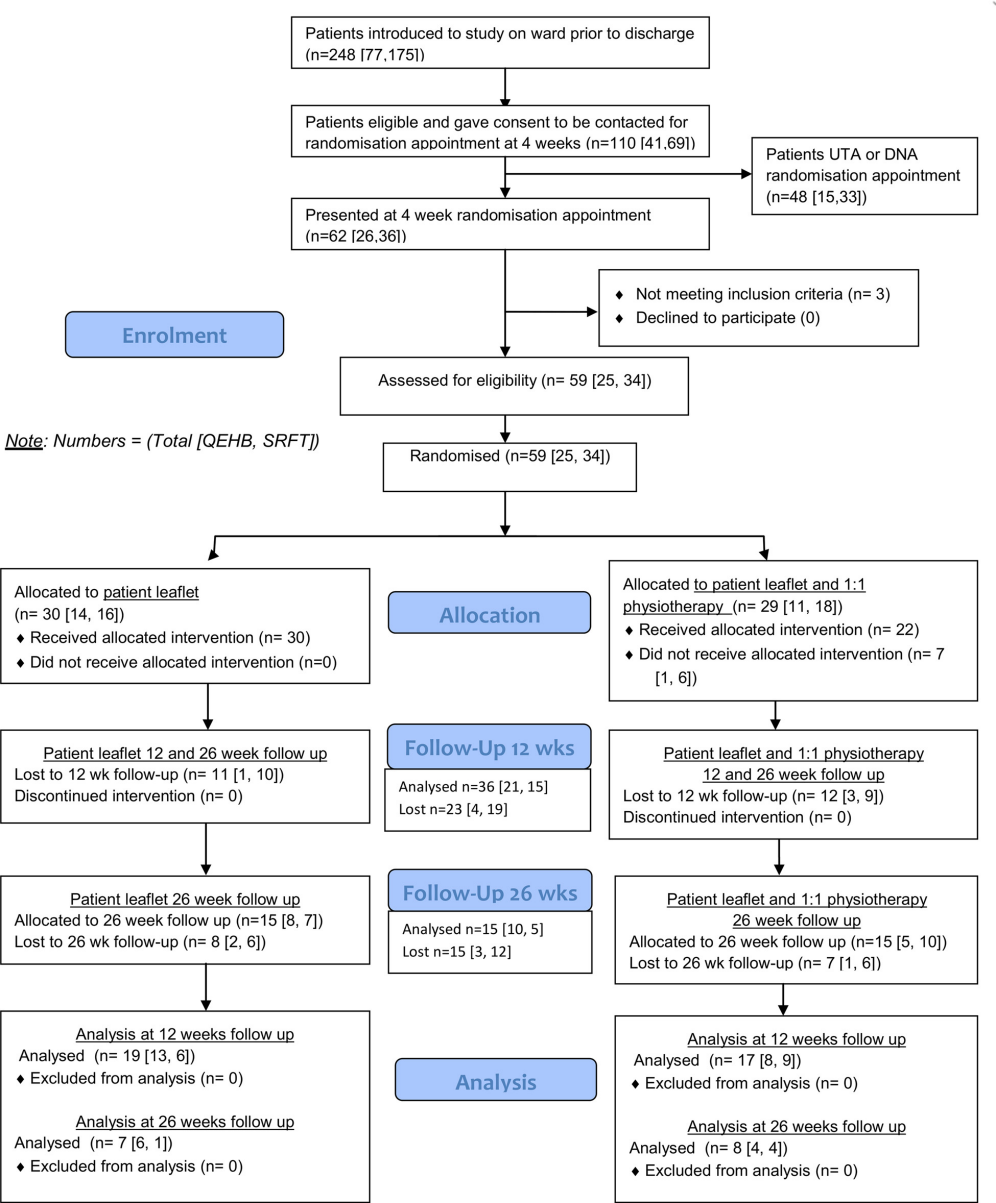
CONSORT diagram (period 14.01.13 to 12.6.14).

The trial ran from 14^th^ January 2013 to 12^th^ June 2014, inclusive of recruitment, outcome assessment and follow up. Fig 1 presents the CONSORT diagram for participant progression through the trial.

### Recruitment

110 eligible patients gave consent to be contacted upon discharge (44% of patients undergoing surgery). At the QEHB site, 41/77 (53%) of introduced patients agreed to be contacted for an appointment at 4 weeks (n = 32 declined, n = 4 not eligible). At the SRFT site, 69/175 (39%) of introduced patients agreed to be contacted for an appointment at 4 weeks (n = 68 declined, n = 38 not eligible). Table 1 details the issues affecting recruitment based on data from the *introducer* physiotherapist. Hospital strategy (waiting list initiatives, management bed pressures) influenced the number of patients available for recruitment, while travel was the key issue for patients not interested in participating. The recruitment factor at the QEHB site (randomised / introduced) was 25/77 (32%), and at the SRFT site was 34/175 (19%).

**Table 1:** Issues affecting recruitment from introducer data.

Category of reasons	QEHB site	SRFT site
Issues affecting recruitment (from physiotherapists’ perspectives)	Surgeon annual leave	3 month period of reduced number of operations because of bed pressures (March-May 2013)
	Waiting list initiatives to private sector	More ineligible patients from May 2013. Possible reasons: rotation Band 5 physiotherapists in May, a difference in the knowledge of the new physiotherapists on the ward, training from the site coordinator, surgeon caseload and type of patients referred
	Staff annual leave	
Reasons for declining (obtained from patients by physiotherapists)	Wanted to attend for physiotherapy	Too far to travel–regional centre with vast catchment area (n = 40)
	Too far to travel (regional centre with vast catchment area)	Organised own physiotherapy (n = 4)
		Has physiotherapy in family (n = 1)
		Did not want physiotherapy (n = 1)
		Thinks physiotherapy increases pain (n = 1)
		No reason given (n = 6)
		Not interested (n = 5)
		Unable to get time off work (n = 5)
		Family commitments (n = 4)
Reasons for ineligibility (from physiotherapists)		Pain (n = 10)
		Co-morbidities (n = 10, 1 was specified as spondylitis)
		Not fluent in English (n = 4)
		Needs post op physiotherapy so unsuitable for randomisation (n = 6)
		Sensory loss (n = 1)
		Lower limb weakness (n = 5)
		Dural tear (n = 2)

Fifty nine (54%) patients were recruited, with n = 3 ineligible at consent/randomisation at 4 week appointment, with reasons of severe leg pain (n = 1), wound haematoma (n = 1), and not meeting criteria (n = 1). Reasons for patients not attending the 4 week recruitment appointment are detailed in Table 2. In addition, n = 6 patients cancelled their 4 week appointment in advance for reasons of: working (n = 1), awaiting MRI results (n = 1), too far to travel (n = 1), and no reason provided (n = 3). The consent rate at the introducer stage was 44% (53% QEHB and 39% SRFT), and at the recruitment stage was 95% (96% QEHB and 94% SRFT). For patients not attending the 4 week recruitment appointment, n = 1 reported that revision surgery was planned, and n = 2 that they had been readmitted.

**Table 2:** Reasons for patients not attending the 4 week recruitment appointment (n = 42).

Reason	QEHB (n = 12)	SRFT (n = 30)
Readmitted to hospital		2
Readmitted for revision discectomy		1
In pain and experiencing rheumatological problems		1
Work or other commitments	1	1
Too far / too difficult to travel	3	1
Confused about appointment and no text reminder received		1
Did not receive appointment details		1
Attended GP surgery by mistake		1
Would participate if surgeon was there as well		1
Did not attend and no reason given / did not respond to follow up contact	3	3
Did not attend on second occasion having been rebooked and no reason given / did not respond to follow up contact	1	3
No longer wishes to participate / no reason provided		4
Advised from elsewhere that needed physiotherapy	1	
Lost appointment details	1	
Caring for mother		1
Daughter off school		1
On holiday during recruitment window	1	
No response to contact for appointment	1	8

### Loss to follow up

Loss to follow up was 39% at 12 weeks and 50% at 26 weeks, with one site contributing 83% losses, suggesting site specific issues e.g. with text reminders for appointments and administrative issues relating to booking appointments at one site. Table 3 details the reasons for losses in situations where it was possible to contact participants by telephone.

**Table 3:** Reasons for loss to follow up at 12 weeks (n = 23) and 26 weeks (n = 15).

**Reason for loss to follow up at 12 weeks**	**QEHB (n = 4)**	**SRFT (n = 19)**
Too far to travel and physiotherapy not helping		1
Did not want to continue as second physiotherapy session made them worse		1
Does not wish to have physiotherapy		1
Readmitted		2
No longer wants to be involved	1	1
Back at work and too far to travel	1	
No reason provided		12
Unable to contact by telephone	1	1
Data sheet lost or did not attend (administrative issue)	1	
**Reason for loss to follow up at 26 weeks**	**QEHB (n = 3)**	**SRFT (n = 12)**
Withdrew at 12 week assessment	2	8
Did not want to come		1
Cold and did not attend 2 reappointments	1	
No reason provided		2
Patient reported that hospital cancelled appointment		1

### Baseline data

Table 4 presents the baseline data by intervention group. Most operations were planned (10% emergencies), and duration of symptoms prior to surgery was considerable (81 months patient leaflet group, 69 months physiotherapy/leaflet group). The median GPE at baseline of 2 illustrates that patients were much improved following the surgery. Most patients were experiencing leg (95%) and back pain (64%) at baseline with mean pain today 2.63 in the patient leaflet group and 2.30 in the physiotherapy/leaflet group reflecting low severity, although 69% participants were taking regular analgesia. Of the n = 36 patients followed up at 12 weeks, n = 33 (92%) had residual symptoms with n = 29 (81%) experienced both leg and back pain. Of the n = 15 patients followed up at 26 weeks, n = 13 (87%) had residual symptoms and all were experiencing both leg and back pain.

**Table 4:** Demographic and baseline statistics by intervention group.

	Leaflet only (n = 30)		Physiotherapy/leaflet intervention (n = 29)	
	n		n	
Age (range, mean ± SD)	30	26 to 64, 44.23 ±11.23	29	26–64, 44.54 ±9.92
Gender (male: female)	30	16, 53	29	12, 41
Employment status	30	Employed 18	28*	Employed 15
		Self-employed 4		Self-employed 8
		Unemployed 4		Unemployed 2
		Retired 3		Retired 2
		Housewife/husband 1		Other 1
Ethnic group	30	White Caucasian 27	28*	White Caucasian 25
		Other white background 2		Other white background 1
		White/black Caribbean 1		Indian 2
Distance from hospital (mean miles ±SD)	30	12.59 ±5.5	29	15.83 ±9.28
Nature of surgery(planned:emergency)	28*	25:3	29	26:3
Duration of symptoms prior to surgery (mean months ±SD)	28*	80.99 ± 143.63	29	68.34 ±93.80
Back pain (number)	30	16	29	22
Leg pain (number)	30	29	29	27
Currently taking pain relief (yes:no)	30	19:11	28*	22:6
RMDQ (range, mean ±SD)	30	1 to 22, 10.07 ±5.58	29	0 to 23, 10.52 ±5.94
GPE (median, interquartile range)	30	2, 1	29	2, 1

* Missing data

### Numbers analysed

For each group, number of participants included in analyses was according to the original assigned groups (Fig 1).

### Acceptability of procedures and interventions

Feedback from researchers and participants supported the efficiency and overall acceptability of trial procedures and interventions. The burden of outcomes assessment was overall acceptable, described as efficient and effective. Some did find the process long but others found they benefitted from the assessment process as it provided feedback on progress. Physiotherapists at one site (Table 1) did perceive that some patients were not willing to be randomised as they wanted to receive the physiotherapy 1:1 intervention.

At 12 weeks 44% (n = 16) assessors correctly guessed the intervention group the participant had been allocated to, 19% (n = 7) guessed incorrectly and data were missing for 36% (n = 13). At 26 weeks, 47% (n = 7) assessors correctly guessed the intervention group the participant had been allocated to, 7% (n = 1) guessed incorrectly, and data were missing for 47% (n = 7).

Data quality for the RMDQ was very good, with 100% of available forms complete at 12 and 26 weeks, and just one missing data point and one unclear data point at baseline. A number of issues were identified with respect to quality and missing data for other outcomes, including: where numerical data were required in text form, in places it was difficult to determine actual value e.g. through clarity of handwriting; for measures where a choice was required more than one value was ticked or no values ticked; there was considerable missing data for the FABQ owing to confusion over the format of the questionnaire that provided an example line for scoring; and due to a photocopying error, the question “prior to your back problem, were you working full or part-time?”, “FABQ” and “Range of Movement” were missing on 3 copies of the baseline assessment forms at the beginning of data collection for 1 site; and the position of the cross on the VAS was not always clear (cross not on the line, type of pen used). Specific physiotherapy interventions were utilised and recorded accurately, but considerable data were missing on intervention sheets. Specifically, free text sections were often left unanswered, and the requested patient discharge summary was provided for 55% participants by the treating physiotherapists. Feedback on staff training at the time was very positive.

Patient data illustrated 100% participants reporting adherence to the advocated exercises at both 12 and 26 weeks. However, the nature of adherence ranged considerably, from at most, participants reporting exercising 3 times per day and others as able to around other activities such as work or gym. Reasons given for reducing exercises included pain, attending spine class, increasing other activities such as golf and walking, cycling, or return to work, and some participants reported ‘exercising in response to days of increased pain’. Other factors which influenced adherence to exercise prescription included motivation.

Two participants reported use of other interventions outside of the trial. At 12 weeks, 1 participant in the 1:1 physiotherapy and patient leaflet group reported having 4–5 sessions of acupuncture that was perceived as beneficial. At 26 weeks, 1 participant in the physiotherapy/leaflet group reported having 6 weeks of weekly 1 hour sessions at a gym.

### Outcomes

Mean (SD) and confidence intervals for the RMDQ are presented in Table 5. 5.1% of zero scores at 12 weeks (patient leaflet only 6.7% and physiotherapy/leaflet 3.4%) and 6.8% of zero scores at 26 weeks (patient leaflet only 6.7% and physiotherapy/leaflet 6.9%) illustrated no floor effect. Sensitivity to change was assessed at 12 weeks with mean (SD) change -4.53 (6.41), 95%CI -7.61 to -1.44 for leaflet; and mean (SD) change -6.18 (5.59), 95%CI -9.01 to -3.30 for physiotherapy/leaflet. RMDQ mean difference (95%CI) between change from baseline to twelve weeks was 1.65 (-2.46, 5.75). Mean difference (95%CI) between groups at 12 weeks was -0.16 (-3.36, 3.04).

**Table 5:** RMDQ data at baseline, 12 weeks and 26 weeks.

Baseline				12 weeks follow-up			26 weeks follow-up		
Intervention	n	Mean (SD)	95% CI	n	Mean (SD)	95% CI	n	Mean (SD)	95% CI
Patient leaflet	30	10.07 (5.58)	8.07 to 12.07	19	5.37 (4.91)	3.16 to 7.58	7	5.71 (7.00)	0.52 to 10.90
Physiotherapy / leaflet	29	10.52 (5.94)	8.36 to 12.68	17	5.53 (4.49)	3.40 to 7.66	8	5.25 (5.55)	1.40 to 9.10

Table 6 summarises descriptively the secondary outcome data (See S1 Minimum data set for raw data). At 12 weeks, 60 and 74% participants had returned to work (physiotherapy/leaflet, patient leaflet groups respectively) and at 26 weeks this rose to 75 and 86% respectively (although based on limited data). Of note is that the reported rates may be underestimates since the question was regarded as not being applicable to 29 and 11% (physiotherapy/leaflet, patient leaflet groups respectively) of participants at 12 weeks and 13 and 14% at 26 weeks.

**Table 6:** Secondary outcome data at baseline, 12 and 26 weeks.

Secondary outcomes			Baseline		12 weeks		26 weeks	
			**n**	**Mean (SD)**	**n**	**Mean (SD)**	**n**	**Mean (SD)**
**VAS Back Pain**	Today	Patient leaflet	30	2.63 (2.00)	19	1.87 (2.56)	7	1.16 (1.47)
		Physiotherapy/ leaflet	29	2.30 (1.80)	17	2.20 (1.65)	8	1.33 (1.22)
	Least in last two weeks	Patient leaflet	30	2.18 (1.76)	19	0.84 (1.05)	7	2.11 (3.34)
		Physiotherapy/ leaflet	29	1.48 (1.31)	17	1.70 (1.60)	8	0.85 (0.91)
	Greatest in last 2 weeks	Patient leaflet	30	5.43 (2.59)	19	3.04 (3.22)	7	3.54 (4.10)
		Physiotherapy/ leaflet	28	4.80 (3.06)	17	4.34 (2.64)	8	2.76 (2.47)
**VAS Leg pain**	Today	Patient leaflet	30	2.71 (2.91)	19	1.67 (2.31)	7	0.67 (1.25)
		Physiotherapy/ leaflet	28	1.62 (2.13)	17	1.74 (2.13)	8	1.83 (2.16)
	Least in last two weeks	Patient leaflet	30	1.80 (1.73)	19	0.80 (0.99)	7	0.54 (0.90)
		Physiotherapy/ leaflet	28	0.84 (1.55)	17	1.79 (2.50)	8	1.41 (1.95)
	Greatest in last 2 weeks	Patient leaflet	30	4.47 (3.53)	19	2.78 (3.11)	7	1.73 (2.58)
		Physiotherapy/ leaflet	28	3.74 (2.93)	17	3.64 (2.82)	8	2.78 (2.26)
**FABQ**	Activity	Patient leaflet	29	12.28 (5.67)	19	8.89 (4.57)	7	8.14 (4.7)
		Physiotherapy/ leaflet	27	13.15 (4.52)	15	11.53 (7.73)	8	11.75 (6.45)
	Work	Patient leaflet	28	17.21 (9.89)	18	14.06 (10.55)	7	16.57 (6.95)
		Physiotherapy/ leaflet	26	19.96 (11.15)	14	16.86 (12.48)	8	13.86 (10.68)
**TAMPA**		Patient leaflet	30	37.53 (10.24)	19	36.47 (7.23)	7	38.00 (13.22)
		Physiotherapy/ leaflet	29	40.48 (6.47)	17	37.35 (8.29)	8	37.88 (7.28)
**Range of movement**	Flexion	Patient leaflet	30	3.82 (2.16)	19	5.34 (3.83)	7	5.17 (1.72)
		Physiotherapy/ leaflet	29	3.54 (1.96)	17	4.47 (1.49)	8	5.00 (2.19)
	Extension	Patient leaflet	30	0.36 (1.86)	19	1.78 (1.24)	6	2.18 (0.67)
		Physiotherapy/ leaflet	29	0.71 (3.40)	17	1.64 (3.96)	8	-0.33 (2.28)
	Left side flexion	Patient leaflet	30	29.55 (18.54)	19	25.23 (12.10)	6	19.42 (4.67)
		Physiotherapy/ leaflet	29	31.56 (17.96)	15	35.59 (17.97)	8	27.19 (16.18)
	Right side flexion	Patient leaflet	30	29.99 (17.83)	17	25.62 (12.37)	6	18.67 (2.42)
		Physiotherapy/ leaflet	29	30.71 (17.89)	15	32.49 (17.38)	8	28.73 (16.27)
**SLR: angle of symptomatic leg**		Patient leaflet	30	68.28 (15.48)	19	78.42 (13.99)	7	70.14 (25.56)
		Physiotherapy/ leaflet	29	66.64 (18.02)	17	80.53 (12.53)	8	84.25 (10.93)
			**n**	**%**	**n**	**%**	**n**	**%**
**SLR of symptomatic leg**	Positive test	Patient leaflet	25	83	9	47	3	43
		Physiotherapy/ leaflet	25	86	9	53	2	25
	Pain	Patient leaflet	16	53	8	42	3	43
		Physiotherapy/ leaflet	14	48	4	24	2	25
	Resistance	Patient leaflet	8	27	8	42	4	57
		Physiotherapy/ leaflet	11	38	9	53	2	25
	Pain and resistance	Patient leaflet	4	13	0	0	0	0
		Physiotherapy/ leaflet	2	7	0	0	0	0
	Missing data	Patient leaflet	2	7	3	15	0	0
		Physiotherapy/ leaflet	2	7	4	24	4	50
**Return to work**	Yes	Patient leaflet	1	3	14	74	6	86
		Physiotherapy/ leaflet	8	28	10	59	6	75
	No	Patient leaflet	26	87	2	16	1	14
		Physiotherapy/ leaflet	19	66	5	30	1	13
	Not applicable	Patient leaflet	3	10	3	16	0	0
		Physiotherapy/ leaflet	1	3	2	12	1	13
	Full time	Patient leaflet	1	3	11	58	6	86
		Physiotherapy/ leaflet	6	21	8	47	5	63
	Part time	Patient leaflet	1	3	3	16	0	0
		Physiotherapy/ leaflet	2	7	2	12	1	13
	Not applicable	Patient leaflet	28	93	5	26	1	14
		Physiotherapy/ leaflet	21	72	7	41	2	25
**Duties on return to work**	Full	Patient leaflet	0	0	11	58	5	71
		Physiotherapy/ leaflet	3	10	7	41	5	63
	Light	Patient leaflet	2	7	3	16	1	14
		Physiotherapy/ leaflet	4	14	3	18	1	13
	No or not applicable	Patient leaflet	28	93	5	26	1	14
		Physiotherapy/ leaflet	22	76	7	41	2	25
**Return to normal activities**	Yes	Patient leaflet	12	40	12	73	4	57
		Physiotherapy/ leaflet	7	24	11	65	5	63
	No	Patient leaflet	18	60	7	32	3	43
		Physiotherapy/ leaflet	22	76	6	32	3	43
			**n**	**Mean (SD)**	**n**	**Mean (SD)**	**n**	**Mean (SD)**
**Return to work: weeks post surgery**		Patient leaflet	1	2.5	14	8.93 (3.39)	6	11.17 (5.08)
		Physiotherapy/ leaflet	8	3.81 (1.60)	10	7.8 (4.71)	5	9.2 (5.54)
**Return to normal activities: weeks post surgery**		Patient leaflet	12	2.45 (1.37)	12	4.81 (3.44)	4	11.5 (4.12)
		Physiotherapy/ leaflet	7	3.21 (1.63)	11	8.18 (4.51)	5	9.4 (3.97)
			**n**	**Mean (SD)**	**n**	**Mean (SD)**	**n**	**Mean (SD)**
**EQ5D Total**		Patient leaflet	30	69.52 (2.23)	19	78.74 (19.53)	7	77.86 (20.59)
		Physiotherapy/ leaflet	29	71.61 (16.50)	17	70.06 (10.58)	8	78.50 (10.43)
			n	**Median (IQR)**	**n**	**Median (Min,Max)**	**n**	**Median (Min,Max)**
**EQSD: Mobility**		Patient leaflet	30	2 (1,3)	19	1.5 (1,3)	7	2 (1,3)
		Physiotherapy/ leaflet	29	2 (1,4)	17	1 (1,3)	8	1 (1,2)
**EQSD: Self-care**		Patient leaflet	30	2 (1,3)	19	1.5 (1,2)	7	1 (1,3)
		Physiotherapy/ leaflet	29	1 (1,3)	17	1 (1,2)	8	1 (1,3)
**EQSD: Usual activities**		Patient leaflet	30	2.5(1,5)	19	1.5 (1,5)	7	2 (1,5)
		Physiotherapy/ leaflet	29	3 (1,5)	17	1 (1,3)	8	1 (1,2)
**EQSD: Pain/ discomfort**		Patient leaflet	30	2 (2,4)	19	3 (1,4)	7	2 (1,3)
		Physiotherapy/ leaflet	29	3 (1,4)	17	2 (1,3)	8	1 (1,3)
**EQSD: Anxiety/ depression**		Patient leaflet	30	1 (1,4)	19	1.5 (1,5)	7	1 (1,3)
		Physiotherapy/ leaflet	29	2 (1,5)	17	1 (1,3)	8	1 (1,2)

### Serious adverse events

No serious adverse events were reported.

## Discussion

### Principal findings

This trial investigated individualised (1:1) physiotherapy management, which is reflective of current practice[13] in several countries including the UK[14]. The participants’ mean age of 44 years was consistent with national data.[5] The median GPE of 2 (IQR 1) at baseline highlights the improvements that patients experienced following surgery supporting reported success rates in the literature,[8] although pain at baseline had only resolved for 2 patients. Although pain was of low severity, 92% participants with residual symptoms at 12 weeks and 87% at 26 weeks is consistent with 30% to 70% of patients experiencing residual pain longer term (1 year).[9] Consistent with the literature, some patients required revision surgery[10] illustrated by the 3 patients unable to attend the 4 week appointment. The results suggest improved return to work data compared to the previously reported 70% fit to return to work 12 months after surgery,[11] and these data are likely to be under-estimates as several participants were not aiming to return to work.

The mean (SD) RMDQ at baseline of 10.07 (5.58) for the patient leaflet and 10.52 (5.94) for the physiotherapy/leaflet group is similar to 14.5 (3.7) for the behavioural graded activity intervention, and 13.5 (4.5) for the usual physiotherapy care group;[42] in a previous trial, in the context that Ostelo et al only recruited patients with persistent problems reflecting their higher baseline values and smaller SD.[42] It was not possible to compare to Danielsen et al[43] who also used the RMDQ, as their baseline was pre surgery. Our mean (SD) change -4.53 (6.41), 95% CI -7.61, -1.44 for leaflet; and mean (SD) change -6.18 (5.59), 95% CI -9.01, -3.30 for physiotherapy/leaflet post intervention at 12 weeks are promising for both interventions (MCID RMDQ 3.5[28]).

Participant adherence with exercises was excellent. Recruitment and consent rates were appropriate. Acceptability of the interventions and procedures was good. Most procedures worked well, but several issues were identified that required further consideration in going forwards to definitive trial (Table 7). There were no adverse events, and the data suggest no evidence of harm and that both interventions may be effective. It is justified to use the RMDQ as the primary outcome in an appropriately powered RCT (using 3.5 points [MCID] on the RMDQ to estimate sample size) as there was no evidence of a floor effect, and mean change RMDQ > minimum detectable change. Recruitment, consent rates for entering the trial, and follow-up rates will be used to estimate feasibility of running the RCT with the existing 5 collaborative sites.

**Table 7:** Issues to further consider in moving to definitive trial.

Issue	Analysis
Loss to follow up	Normal practice at SRFT was not to see patients routinely for physiotherapy and this may have implicitly contributed to the high losses to follow up and the 6 patients not attending for the physiotherapy/leaflet intervention to which they were allocated (only n = 1 for QEHB).
	Follow up reminders were different between sites (text versus telephone). SRFT with the high loss to follow up, used a text reminder system and it was hypothesised that this may be contributing to the losses. However, changing this to telephone reminders for the final third of SRFT follow ups did not make a difference.
Outcome assessment	Telephone follow up of the primary outcome measure would be valuable as the physiotherapists perceptions of distance to travel and returning to work as key factors for loss to follow up are also reflected in the secondary outcome data; with 60% (physiotherapy/leaflet) and 74% (patient leaflet) back at work at 12 weeks, and 75% (physiotherapy/leaflet) and 86% (patient leaflet) back at work at 26 weeks.
Training	The data suggest that there may have been a training issue at SRFT regarding *assessor* checking for missing data, although training was consistent across sites. This may therefore have been a reflection of demands on busy clinicians, as it was also true of the intervention data that in particular had not included any data in free text sections for SRFT. All submitted forms from QEHB included a detailed summary at discharge, whilst the discharge summary was briefly outlined on only 1 form from SRFT. It would be valuable to increase the monitoring at each site regarding the data completion of outcome measures and intervention record data across both sites.
Performance based outcomes	The value of the performance based outcomes, the SLR and ROM lumbar spine, is unclear from this data considering the cost of physiotherapist time to collect data, and patient attendance for outcome assessment.

### Strengths and weaknesses

This trial achieved its objectives and provided important data to inform a definitive trial. The sample size was purposely small owing to the focus of the trial objectives, but the losses to follow up were higher than anticipated. Attrition rates across existing trials vary and were unclear for many.[8,14] They ranged from no losses in either arm[44] to 32% and 33% losses in the control trial arms.[45,46] Most losses were however <20%.[47] This trial’s losses were therefore higher than existing trials (39% at 12 weeks and 50% at 26 weeks), but when explored by site, losses for QEHB were acceptable (7% and 10% at 12 and 26 weeks respectively); and losses for SRFT unacceptable (32% and 40% at 12 and 26 weeks respectively). There is a lack of data regarding population characteristics of participants who fail to complete trials investigating rehabilitation post spinal surgery, but these data provide insight into travelling distance and returning to employment.

### Implications

Based on these results, a RCT would be feasible. Moreover, it is essential[8,14] with a revised design. The potential to improve disability in this population was seen, as there was an improvement in RMDQ at 12 weeks in both groups, and the mean change in both groups was greater than the MCID. It is therefore plausible that both physiotherapy/leaflet intervention and patient leaflet alone have the potential to improve disability. Both arms of this trial can therefore be considered as active interventions designed to improve outcomes post surgery if the improvement seen in both arms is greater than the normal course of recovery. Prospective cohort studies evaluating disability 0–3 months following surgery support a plateau of improvement at 4–6 weeks.[48,49] The data support a stepped care intervention[50] combining patient leaflet and physiotherapy intervention compared to no intervention. Some patients are likely to respond to the patient leaflet as the first low intensity ‘step’ while others may need a more intensive step to physiotherapy intervention. The integration of patient preference and physiotherapist clinical reasoning into a stepped care model reflects current trends in the NHS. Both clinical sites are currently considering a model of patients opting in to attend for physiotherapy management, therefore capturing patient preference, and avoiding wasted resources through patients not attending booked appointments. Very different definitions of recovery are often used in the literature and it is therefore difficult to obtain pooled estimates of recovery rates. This places emphasis on our detailed understanding of the natural clinical course of disability and other key outcome measures over time post lumbar discectomy. To fully inform the components of the stepped care intervention, further data is required to fully understand 1] the natural clinical course of disability and other key outcomes, and 2] the prognostic factors indicative of poor outcome post first time lumbar discectomy.

### Future research

Further consideration of trial design and interventions is required. The exploratory analysis in this trial found a mean difference (95% CI) between groups at 12 weeks of -0.16 (-3.36, 3.04). The trend within the results was to support the potential value of both interventions. This contrasts to previous trials that suggested improvement of active rehabilitation over patient information.[45,51–54] This trial did not compare to a 'no intervention group' control and can therefore offer no conclusion as to whether either patient leaflet or physiotherapy/leaflet may be more beneficial than 'normal' recovery; a feature of pragmatic trial design. The patient leaflet for this trial was developed as no surgery specific leaflet existed, and this may have enhanced its potential effectiveness, therefore negating our hypothesised preliminary superior efficacy of the physiotherapy/leaflet intervention. This small mean difference is similar to comparisons in existing trials using the RMDQ. Danielsen et al[43] found a mean difference (95%CI) of -1.00 (-4.27 to 2.27) when comparing high and low intensity exercise programmes long term,[8] and Ostelo et al[42] found a mean difference of 0.4 (–1.8 to 2.6) comparing behavioural graded activity intervention to usual physiotherapy care at 3 months (immediately post intervention). In both trials[8,42] the change within groups suggested potential benefit of both interventions, highlighting the importance of careful trial decisions regarding comparator interventions.

## Conclusions

In conclusion, both interventions were acceptable. It is promising that both interventions demonstrated a trend in reducing disability in this population. A RCT, using a different trial design, is needed to ascertain the effectiveness of combining the interventions into a stepped care intervention and comparing to a no intervention arm. All findings from this pilot and feasibility trial will guide changes to improve acceptability for the RCT. The RCT should consider also long term follow up of patients outcome, where losses to follow up would be a key consideration.

## Supporting Information

S1 Protocol (DOCX)

S1 Minimum data set (XLSX)
