# Supplementary material for: Physiotherapy Post Lumbar Discectomy: Prospective Feasibility and Pilot Randomised Controlled Trial
Source: PLoS One. 2015 Nov 12;10(11):e0142013. doi: 10.1371/journal.pone.0142013 (PMC4642943; doi:10.1371/journal.pone.0142013)
Supplement: S1 Protocol — (DOCX) [file pone.0142013.s001.docx]

| 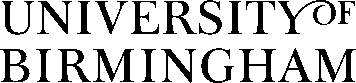 | 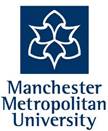 |
| --- | --- |

1. **Title of Project**

QUeen Elizabeth physiotherapy post lumbar discectomy STudy: pilot work to improve service delivery and inform the development of a randomised controlled trial - QUEST

1. **Chief Investigator (CI)**

Rushton A., EdD, Senior Lecturer Physiotherapy, College of Medical and Dental Sciences, University of Birmingham (UoB).

1. **Principal Grant Holder**

Heap A. Extended Scope Physiotherapist Spinal Surgery, Physiotherapy Department, Queen Elizabeth Hospital Birmingham (QEHB)

1. **Funding authority**

Queen Elizabeth Hospital Birmingham Charity. £12,000 (over 18 months)

1. **Co – investigators**

Barrett T., Clinical Lead, Physiotherapy Department, Salford Royal NHS Foundation Trust (SRFT)

Bayliss C., patient representative, QEHB

Calvert M., PhD, Reader, College of Medical and Dental Sciences, UoB

Goodwin P., PhD, Senior Lecturer Physiotherapy, Faculty of Health, Psychology & Social Care, Manchester Metropolitan University (Lead at SRFT)

Hamid N., Mr, Consultant Neurosurgeon, QEHB

Littleford C., patient representative, QEHB

Paul E., Clinical Lead, Physiotherapy Department, SRFT

White L., Extended Scope Physiotherapist- Spinal Surgery, QEHB

Wright C., Senior Lecturer, College of Medical and Dental Sciences, UoB

1. **Principal Investigators (PI)**

Goodwin P. at SRFT

Rushton A. at QEHB

**User Involvement**

For this study, users are patients who have undergone recent first-time lumbar discectomy surgery (including microdiscectomy) through QEHB (the lead site) or SRFT (second site). User involvement is very important and has been built into each stage of the study following advocated approaches for user involvement in research in general (Staley, 2007) and, more specifically, in low back pain (Ong and Hooper, 2003). Care has been taken to involve users with ethnic diversity to broaden applicability of study findings. Two users are members of the Study Management Group (SMG) and have informed, for example, study procedures, choice and administration of outcome measures, and content of Patient Information Sheets. A user representative will sit on the Study Steering Group (SSG). In addition, users have participated in focus groups to inform the content and acceptability of the two interventions to be assessed in this study. Following completion of their involvement in the study, 10 to 12 participants will participate in each of two focus groups at each study site to provide feedback on acceptability of procedures and interventions. Involving users in all stages of the study should ensure that the research is relevant to patients.

The Chief Investigator (CI) will provide training, mentorship and guidance for all users. This will include informal (e.g. conversations before and/or after meetings) and more formal training sessions (e.g. induction to and training on the research process). It is anticipated that there will be three to seven meetings (SMG and SSG) per year. Some meetings will be local to Birmingham (at QEHB or UoB) and some will take place via conference call using home or work telephone numbers. Each meeting will last around 2 to 3 hours. Funding for user travel has been obtained to support costs incurred by users to attend meetings.

1. **Background**

Low back pain affects 80% of the population within their lifetime (WHO, 2003) and contributes to lost productivity and sickness/disability benefit estimated at £10,668million annually (Maniadakis & Gray, 2000). Inpatient surgical treatment is the largest single component of expenditure in managing this condition. In 2008/9, 9677 lumbar discectomies were performed within the NHS for a primary indication of leg pain (HESonline, 2010). Post-operative rehabilitation is a key issue, with 30% to 70% of patients experiencing residual pain (Raymond et al, 2004), 3% to 12% seeking further surgery (CBO, 2008), and only 70% fit to return to work 12 months after surgery (Donceel & Du Bois, 1998).

A UK audit of spinal surgeons (McGregor et al, 2006) identified that post-operative advice was variable. For example, the period recommended for 'no sitting' ranged from 2 to 42 days, and routine referral of patients for physiotherapy after discharge was made by 55% of surgeons. A survey of physiotherapy management post lumbar discectomy (Williamson et al, 2007) identified that individual, out-patient physiotherapy was provided routinely in 44% of spinal centres and in another 46% of centres for patients who had residual problems, such as pain. The content and advice provided were very variable e.g. number of sessions ranged from 1 to 20, and a wide range in type of exercises was prescribed. The surveys raised questions about the need for research to optimise rehabilitation for this patient group.

A Cochrane systematic review of group exercise programmes post lumbar disc surgery (Ostelo et al, 2008) included trials to May 2007. The quality of included trials was variable, with seven of the 14 included in the review being evaluated as a low risk of bias. Low to moderate evidence supported exercise as being more effective than no treatment, and high intensity exercises as being more effective than low intensity for pain and improved physical impairment. There are some limitations of the review, in particular, that the surgery included a broad range of approaches – standard discectomy, microdiscectomy, laser discectomy and chemonucleolysis. In addition, duration and content of management was variable, and individualised physiotherapy was not investigated. There is disagreement, therefore, between the management included in the review (Ostelo et al, 2008) and current practice in the UK (Williamson et al, 2007).

A more recent systematic review (Rushton et al, 2011) evaluated effectiveness of physiotherapy intervention post first single level lumbar discectomy at 3 months (short-term) and 6 months (long-term), on clinically relevant outcomes of disability, function and health. All physiotherapy outpatient interventions were included. The majority of trials involved group rehabilitation. Three trials investigated individualised (1-1) physiotherapy management, which is reflective of current practice (Williamson et al, 2007) in several countries including the UK. The review identified only one of 16 trials as having low risk of bias, seven as unclear and eight as high risk of bias. Some evidence supported that physiotherapy improved disability in the short-term, with a potential benefit of more intensive intervention. Weak evidence supported improved movement and physical impairment, in the short-term. Overall, there was inconclusive evidence for effectiveness of outpatient physiotherapy post first lumbar discectomy. The need was indicated for further evidence to inform best practice for this patient group.

There is minimal evidence to support existing physiotherapy interventions that are used post lumbar discectomy in the NHS. Additionally, it is unclear what constitutes best post-operative care. A clinical trial is required, therefore, to identify whether best practice 1:1 physiotherapy is more effective than education alone, and whether it would be a cost-effective use of resources. Not addressing this problem could result in increased societal costs and unhelpful illness behaviour (Linton, 2000), leading to reduced quality of life, as well as, increased NHS costs in terms of additional GP, physiotherapy and consultant appointments, and further surgery.

Use of a standardised, evidence based Patient Leaflet and optimised evidence based individualised (1:1) physiotherapy intervention could improve patient outcome, providing enhanced care for optimal recovery and socio-economic benefit. The following proposed study will provide data to inform changes to the design and conduct of a multi-centre randomised controlled trial (RCT) to evaluate the effectiveness of 1:1 physiotherapy outpatient management (including Patient Leaflet) compared with use of a Patient Leaflet alone following lumbar discectomy.

1. **Research Question and Aims**

Based on findings from a comprehensive systematic review (Rushton et al, 2011), the proposed research question for the RCT is:

What is the effectiveness and cost-effectiveness (MacPherson, 2004) of 1:1 physiotherapy outpatient management (including Patient Leaflet) compared with use of the Patient Leaflet alone following lumbar discectomy, using a validated measure of disability?

The research presented in this protocol is a feasibility and external pilot study (referred to as ‘Study’ throughout). It has been designed to provide data to inform changes to the design and conduct of the RCT. If findings indicate that some aspect within the Study is unacceptable to a large number of participants, the personal and financial consequences would be minimal compared with execution of a RCT.

The specific aims of this Study are:

1. Can this study be done?
2. What are the estimated values for the mean and standard deviation of the proposed primary outcome measure, at the beginning of the study and of change at 12 weeks follow-up?
3. Do the separate components of the study work together (e.g. recruitment, randomisation, analysis) and run smoothly (e.g. follow up assessments)?
4. **Objectives**

Objectives of the Study are to:

1. Evaluate acceptability of interventions to patients, to inform potential modifications.
2. Evaluate the distribution of scores on the Roland Morris Disability Questionnaire (RMDQ, primary outcome measure on disability) for use in the targeted population to:
   1. Inform its appropriateness, in particular with regards to potential floor effects (Arain et al, 2010).
   2. Estimate the standard deviation of scores to inform the sample size calculation for an adequately powered RCT (Arain et al, 2010).
3. Assess feasibility of acquiring the required sample size in a realistic time-scale, for a RCT (Arain et al, 2010).
4. Evaluate acceptability and feasibility of individual Study procedures, to optimise them for a RCT, including:
   1. Recruitment strategy, in particular, acceptability to participants and recruiters (Lancaster et al, 2004; Shanyinde et al, 2011).
   2. Eligibility criteria (Lancaster et al, 2004).
   3. Randomisation to the two interventions (acceptability of the process to participants and recruiters) (Lancaster et al, 2004; Shanyinde et al, 2011).
   4. Blinding procedures (Lancaster et al, 2004; Shanyinde et al, 2011).
   5. Data collection (including use of data collection forms) (Lancaster et al, 2004).
   6. Follow up procedures at 4 weeks post surgery (baseline) and, 12 and 26 weeks post baseline (Lancaster et al, 2004; Shanyinde et al, 2011).
5. Appraise the best way of providing information to patients to enable them to make an informed decision about participating in the RCT.
6. Evaluate:
   1. Consent rate for patients entering the Study (Ross-McGill et al, 2000 Shanyinde et al, 2011).
   2. The training provided for the different roles that physiotherapists undertook in the Study.
   3. Participant compliance rates (Arain et al, 2010).
7. Determine whether different procedures used in the Study work together (Shanyinde et al, 2011).
8. Assess proposed procedures for data analysis (Lancaster et al, 2004).
9. **Study Design**

This study is a feasibility and external pilot study to inform a multi-centre RCT that will compare overall effectiveness and cost effectiveness (MacPherson, 2004) of two interventions, delivered in the outpatient setting, for patients post first lumbar discectomy. Assessments will be made at 4 weeks post surgery (baseline assessment) and 12 weeks post baseline (the proposed primary end point for the RCT). In addition, 50% of the participants will be followed up at 26 weeks to evaluate short-term follow up.

- 1. **Design**

The Study will be a small-scale randomised controlled trial, with random allocation to two interventions, 1:1 physiotherapy outpatient management (including Patient Leaflet) and Patient Leaflet alone.

Following their participation, two focus groups will be conducted (Kitzinger, 1995 & 2006) with participants at each site to explore acceptability and feasibility of the two interventions. Additionally, one focus group will be held at each site with Study physiotherapists.

The Study will be conducted in line with GCP (ICH E6 2006; ICH E9 2006).

- 1. **Procedures and Training**

At each Study site, physiotherapists who are part of the research team will be allocated to the following four roles – *Introducer*, *Recruiter*, *Assessor* or *Treating* physiotherapist. The Flow Diagram in Section 12 gives an overview of stages in the study and responsibility of each role. The two site specific leads will deliver on-site, role-specific training sessions for the physiotherapists to standardise procedures associated with each role.

*Introducers* will invite patients to participate in the Study at their pre-admission visit or discharge session (current practice varies across sites). The *Introducers* will discuss the contents of the Participant Information Sheet and the Patient Leaflet with patients who express an interest in participating in the Study, and obtain written consent to contact the patient to arrange an assessment appointment.

*Recruiters* will discuss the Patient Leaflet with patients at the assessment appointment and answer any questions. They will assess eligibility of patients who are willing to participate and obtain formal, written consent from the eligible patients (called participants). Separate consent will be obtained for participation in the focus groups. *Recruiters* will obtain the group to which each participant is randomised and whether the participant would need to attend hospital for an assessment at 12 weeks or at both 12 and 26 weeks. *Recruiters* will ask each participant not to discuss their treatment with *Assessors* (who will be identified by name) and explain the importance of this request.

*Assessors* will conduct participant assessments at baseline, 12 weeks post baseline and, depending on the group to which the participant was allocated, at 26 weeks post baseline. They will not have access to previous assessment sheets. At the start of each assessment, *Assessors* will remind participants not to talk about their treatment. *Assessors* will be masked to allocation and **will not take part in recruitment, allocation or treatment processes**.

*Treating* physiotherapists will be trained in the use of standardised procedures to inform their use of the Patient Leaflet and 1:1 physiotherapy. The Study sites may adapt the procedures to accommodate differences in practice/facilities locally, as recommended in the MRC guidance for developing and evaluating complex interventions (MRC 2008).

- 1. **Randomisation – Allocation Concealment**

Consecutive consenting patients across the two sites will be randomised to one of the two interventions using a secure computer programme. A few questions will be used to ensure participant eligibility before assigning group allocation. Stratification (Treasure and MacRae, 1998; Moher et al., 2012) will occur based on scores of RMDQ (<15 and ≥15 (Vlayen et al 1995)). Allocation will have equal weighting to the two intervention groups and 50% follow-up at 26 weeks per group. Potential participants and *Recruiters* will be blind to treatment allocation prior to the point of allocation.

- 1. **Blinding**

The nature of the two interventions prohibits blinding participants and treating physiotherapists to the allocated treatment. This is a well recognised limitation to controlling potential sources of bias when evaluating complex interventions (Roland & Torgerson, 1998; Stephenson & Imrie, 1998).

- 1. **Setting**

The setting will be two outpatient Physiotherapy departments – Queen Elizabeth Hospital Birmingham (QEHB, the primary site for the Study) and Salford Royal NHS Foundation Trust (SRFT). The use of two sites should ensure that the required number of participants is acquired in a timely manner. These sites reflect areas of ethnic diversity, consistent with collaborative sites (five at present) for the RCT. Sensitivity to cultural differences will be addressed more fully in the protocol for the RCT, as well as full costing for translation of all documentation. Charity funding granted for this study does not cover translation of the Patient Leaflet. Support for the Study has been agreed by therapy managers and spinal surgeons at each site.

- 1. **Participants**

Inclusion Criteria: male and female patients aged >18 years; post primary, single level, lumbar discectomy (including microdiscectomy) (Selkowitz, 2006), able to communicate in English.

Exclusion criteria: previous surgery at the same spinal level; co-morbidities that might impact on ability to participate in study interventions such as neurological disorders, cognitive dysfunction, uncontrolled cardiovascular disease (Selkowitz et al, 2006), osteoporotic fracture, spondylolythesis, MS, tumour (Foster, 2007); complications from surgery such as excessive bleeding, severe intra-operative root damage, level error, or severe wound infection (Barrios et al, 1990; Selkowitz et al, 2006) that would prevent participation in either intervention; and participation in a concurrent trial.

- 1. **Participant recruitment**

Patients will be invited to participate in the Study at their pre-admission visit or prior to discharge from hospital following surgery (current practice varies across sites). Patients who are interested in participating in the Study will be given a copy of the Participant Information Sheet. An *Introducer* will discuss the Study with them and answer any questions. The *Introducer* will check eligibility of patients who are still interested in participating and request written consent from eligible patients to be contacted with the date/time of an out-patient appointment, 4 weeks post surgery. The *Introducer* will give the patient a Patient Leaflet and discuss this, answering any questions from the patient.

A potential participant will NOT be eligible for inclusion if they have a definite contra-indication to the treatment, as judged against the eligibility criteria through a standardised screening procedure. With their consent, participants’ GPs will be notified of their participation in the Study.

- 1. **Interventions**

The two interventions are: (1) individualised (or, 1-1) physiotherapy and the Patient Leaflet; and, (2) the Patient Leaflet alone.

The 1-1 physiotherapy and the Patient Leaflet were developed through a rigorous process. The interventions were circulated to users, physiotherapists and surgeons at the two Study sites and to the three other collaborative centres for their agreement regarding potential acceptability for implementation as part of the proposed RCT. Care was taken throughout to include participants from ethnic diverse backgrounds to be representative of the target population for the interventions.

1-1 physiotherapy

The 1-1 physiotherapy intervention encompasses education, advice, mobility exercises, core stability exercises, a progressive approach to exercise and encouragement of early return to work and activity; with patients attending up to 8 physiotherapy sessions, over a period of up to 8 weeks (to allow for patient choice and variations in practice at each trial site), starting at approximately 4 weeks post surgery to provide optimal care. It incorporates flexibility for physiotherapists to tailor management to individual patients, thereby ensuring patient centred practice, in line with MRC guidance for developing and evaluating complex interventions (Craig et al, 2008).

The intervention has been designed to reflect best practice, based on evidence in McGregor et al (2007; Williamson et al (2007), Ostelo et al (2008) and Rushton et al (2011). It was developed and agreed by the research team, clinical experts and spinal surgeons (at the five spinal centres, planned as sites for the RCT), physiotherapists and patients.

Patient Leaflet

No surgery-specific leaflet existed nationally; therefore, the Patient Leaflet was developed through a 3-round Delphi study, from information on existing leaflets at the five spinal centres and focus groups involving patients and clinicians. The Delphi study used a purposive sample (n=51) of experts including spinal surgeons, inpatient and outpatient physiotherapists and lumbar discectomy patients from the five collaborative centres for the RCT. The Patient Leaflet includes sections on: anatomy, disc herniation, surgery, activity post surgery, exercises and their progression, and frequently asked questions.

- 1. **Assessments**

Assessments will take place in physiotherapy clinics, with participants being scheduled from across the two interventions per clinic. Baseline assessment of participants will be made following written consent to participate in the Study, at 4 weeks after surgery, and post intervention assessment at 12 weeks post baseline. Follow-up assessment at 26 weeks post baseline will be made on 50% of participants in each intervention group.

Assessments will be recorded on a standard sheet and will comprise patient reported and performance based outcome measures. Assessment sheets will be stored locally in locked cabinets at each site and will be forwarded to the CI upon completion of data collection, to be stored in a locked cabinet at UoB.

- 1. **Outcome Measures**

Completion of all outcome measures is expected to take a maximum of 30 minutes at baseline (including demographic data), and a shorter time period at follow-up(s).

Demographic Data:

Demographic data (to include age, gender, duration of symptoms prior to surgery, nature of symptoms e.g. (leg /back pain, neurological symptoms), occupation, ethnicity and post code will be collected to describe participant characteristics.

Primary Measure

The primary outcome for the RCT is planned to be the Roland Morris Disability Questionnaire (RMDQ), an extensively used disease-specific measurement tool for low back pain, with established properties of reliability and validity (Deyo et al, 1998). Evidence indicates good discrimination for patients with mild to moderate disability and pre-eminence for post-lumbar disc surgery (Ostelo et al, 2004). Minimum clinically important change is reported as 3.5 points (Ostelo and de Vet, 2005). The RMDQ is a 24 item scale, scored 0-24 with 0 indicating no dysfunction and completion takes 5 minutes. The tool will be self-administered by participants at baseline and follow-up(s) and, with their prior consent, telephone-administered for non –attendees at 12 weeks follow-up.

Secondary Measures

The choice of secondary measures has been informed by patients involved in the development of this research, surgeons who consented to access to their patients and physiotherapists, to ensure their importance to patients. The primary aim was not to overburden participants, whilst collecting data that covered all important outcomes post lumbar discectomy (i.e. body function, body structures, activities and participation, and environmental factors (in accordance with the International Classification of Functioning, Disability and Health) (WHO 2001). The selected tools include performance based measures and patient-reported measures. Each was used in at least one study reviewed in our recent systematic review on rehabilitation post first-time lumbar discectomy (Rushton et al, 2011).

The most important secondary outcome is the Global Perceived Effect scale (GPE). This is a self-report measure of patient’s perceived effect of treatment (Beurskens et al, 1996; Ostelo and de Vet, 2005). It is rated 1 to 7, where 1=completely recovered, 2=much improved, 3=slightly improved, 4=not changed, 5=slightly worse, 6=much worse and 7=worse than ever compared with pre-surgery.

Other secondary measures are:

1. VAS back pain and VAS leg pain (0 to 10cm, with 0 labelled as “No pain” and 10 as “Worst pain ever”) (Ostelo & de Vet, 2005); both reported for “today”, “least level of pain over the past 2 weeks”, and “greatest level of pain over the past 2 weeks”.
2. Straight Leg Raise (cm).
3. Time to return to work / normal function /full duty (as relevant) from date of operation.
4. EQ-5D 5L–a patient-reported outcome being implemented into routine care within the NHS.
5. Tampa Scale for Kinesiophobia (fear of movement); a 17 item scale, each item rated as 1 “strongly disagree”, 2 “disagree” , 3 “agree”, or, 4 “strongly agree”.
6. Fear avoidance and beliefs questionnaire.
7. Range of lumbar movement.
8. Use of medication.
9. Re-operation.

In addition, at their final assessment (at 12 or 26 weeks post baseline), every participant will be asked to indicate:

1. Level of compliance with home exercises recommended in the Patient Leaflet, and, for participants in the 1:1 physiotherapy group, level of compliance with any additional home exercises recommended by the physiotherapist.
2. Use of physiotherapy or other intervention (e.g. chiropractic) outside the Study – as an indication of potential dilution of treatment effect if taken up by participants in the Patient Leaflet only group; or, as an indication of potential inflation effect if taken up by patients in the 1-1 group.

Losses to follow-up will be monitored at 12 and 26 weeks post baseline.

- 1. **Sample size**

Based on information in Browne (1995) and Hertzog (2008), thirty patients would be required in each intervention arm at the 12 week assessment (primary end point of RCT). A total of 70 patients will be recruited to allow for attrition (with equal randomisation across groups).

- 1. **Process/Data analysis**

1. Patients’ acceptability of the two interventions and individual procedures (recruitment strategy, application of eligibility criteria, consenting process, randomisation procedure, blinding procedures, data collection, assessments, follow-up procedures, and training researchers and physiotherapists in Study tasks) will be assessed through two focus groups (one for each intervention), at each site. Feedback from physiotherapists (all roles within the Study) will be assessed through one focus group at each site. Formal written consent for participation at a focus group will be obtained from each participant at the beginning of the meeting, following explanation of the purpose of the focus group and what participation would entail.

Data will be analysed following guidance in Kitzinger (1995, 2006). Findings will guide changes to improve acceptability for the RCT. If there are serious concerns about acceptability of the interventions and/or individual procedures, the RCT will not be conducted.

1. Patients’ and physiotherapists’ perceptions of how well different procedures worked together will be appraised alongside acceptability (i above). Findings will guide changes to improve management of the RCT.
2. RMDQ is the proposed primary outcome for the RCT.

The distribution of scores in the Study will be considered at baseline, at 12 weeks follow-up, and for change from baseline to 12 weeks. The % of zero scores at 12 weeks follow-up will be used as the measure of a potential floor effect. Evidence of a large floor effect would cast serious doubt on the choice of RMDQ as the primary, disease-specific, measurement tool in the RCT.

Assuming no evidence of a large floor effect, baseline data and change scores of RMDQ at 12 weeks will be used to estimate the standard deviation (and 95% confidence intervals) for scores and change scores, respectively. These values will support the calculation of sample size for the RCT. In addition, estimated values for the mean and standard deviation will be compared with values published in low back pain studies (Ostelo et al, 2004; Rushton et al, 2011).

1. Recruitment, consent rates for entering the Study, and follow-up rates will be calculated for QEHB and SRFT. These will be used (with the estimated range of sample sizes) to estimate feasibility of running the RCT with the existing five collaborative sites.
2. Participant compliance with treatment will be monitored through data collected at assessments at 12 and 26 weeks follow-up. If compliance is found to be very low, the RCT will not be conducted.
3. The primary outcome (RMDQ) will be assesed using the proposed method for the RCT, using a mixed model with sites as random effects and treatment as a fixed effect, accounting for baseline RMDQ and duration of leg pain. Similar analyses will be used for relevant secondary outcomes. GPE will be dichotomised (scores <3, scores ≥3; Ostelo & de Vet, 2005) prior to analysis and appropriate non linear models developed accordingly.
4. **Flow diagram of Feasibility and Pilot**

**At pre-admission visit or discharge session, *Introducer*:**

- Introduces the study to the patient
- If patient is interested, gives them the ’Participant Information Sheet’
- Answers any questions & ascertains continuing interest in participation
- If yes, checks patient’s eligibility
- If yes, asks patient whether they are willing to be contacted to attend an assessment appointment 4 weeks post surgery
- If yes, obtains written consent to contact patient to arrange an outpatient assessment appointment at 4 weeks post surgery
- Gives patient the ‘Patient Leaflet’, takes them through it & answers any questions

not interested /

not willing to attend /

written consent not given

unwilling to attend

interested, willing to attend AND written Consent given

Patient follows routine procedure at site

**Patient Leaflet**

**only**

ineligible

***Assessor:*** Follow-up assessments

- 50% at 26 weeks post baseline

***Recruiter*** thanks patient for their willingness to participate & explains the ineligibility

**Study consent, randomisation, and baseline assessment appointment (week 4):**

***Recruiter***

- Discusses any issues related to the study and Patient Leaflet
- Answers any other questions
- Ascertains patient’s continued interest in participation
- If yes, determines patient’s eligibility for participation
- If yes, obtains written consent for participation and assigns study ID number

***Assessor:*** Follow-up assessment

- 100% at 12 weeks post baseline

***Assessor:*** Follow-up assessments

- 50% at 26 weeks post baseline

randomisation

***Assessor:* also at 4 week visit**

- Conducts baseline assessment & arranges date for 12 week visit

***Recruiter*:**

1. Obtains randomised group & end point (12 or 26 weeks)
2. Notes participant ID, study group etc
3. Gives information to participant
4. Forwards details to Administrator to write referral letter to GP

eligible

**Patient Leaflet +**

**Individual physiotherapy**

***Treating*** physios

***Recruiter*** thanks patient for their attendance

not interested /

written consent not given

unwilling to attend

At each site: two participant & one physiotherapist focus groups for feedback on acceptability/experience of interventions.

Written consent taken at each focus group.

Patient follows routine procedure at site

***Assessor:*** Follow-up assessment

- 100% at 12 weeks post baseline

**Proposed Timetable**

The duration of the Study will be 18 months, with participant recruitment taking place over 7 months, based on projected throughput numbers obtained from both sites (QEHB and SRFT).

**Start Date for Study:** January 2013

| **Task timeline in months** | **pre** | **1** | **2** | **3** | **4** | **5** | **6** | **7** | **8** | **9** | **10** | **11** | **12** | **13** | **14** | **15** | **16** | **17** | **18** |
| --- | --- | --- | --- | --- | --- | --- | --- | --- | --- | --- | --- | --- | --- | --- | --- | --- | --- | --- | --- |
| 1. Identify personnel at both sites |  |  |  |  |  |  |  |  |  |  |  |  |  |  |  |  |  |  |  |
| 1. Obtain ethical approval |  |  |  |  |  |  |  |  |  |  |  |  |  |  |  |  |  |  |  |
| 1. Identify Study Administrator/ Coordinator (research support teams UoB). |  |  |  |  |  |  |  |  |  |  |  |  |  |  |  |  |  |  |  |
| 1. Complete management protocols for patient recruitment (including screening tool), consent, randomisation, assessment. |  |  |  |  |  |  |  |  |  |  |  |  |  |  |  |  |  |  |  |
| 1. Train Study personnel at 2 sites – informers, recruiters, assessors & treating therapists. |  |  |  |  |  |  |  |  |  |  |  |  |  |  |  |  |  |  |  |
| 1. Recruit & randomise participants at both sites. |  |  |  |  |  |  |  |  |  |  |  |  |  |  |  |  |  |  |  |
| 1. Collect data on outcome measures at both sites |  |  |  |  |  |  |  |  |  |  |  |  |  |  |  |  |  |  |  |
| 1. Conduct data entry, cleaning & preparation for analysis. |  |  |  |  |  |  |  |  |  |  |  |  |  |  |  |  |  |  |  |
| 1. Conduct patient, & physiotherapist, focus groups – acceptability & experience of two interventions | Two Patient focus groups at 2 sites | | | | | | | | | | |  |  |  |  |  |  |  |  |
|  | Physiotherapist focus groups at 2 sites | | | | | | | | | | | | | |  |  |  |  |  |
| 1. Analysis data from focus groups and meetings |  |  |  |  |  |  |  |  |  |  |  |  |  |  |  |  |  |  |  |
| 1. Conduct data analysis from Study |  |  |  |  |  |  |  |  |  |  |  |  |  |  |  |  |  |  |  |
| 1. Write Study report for QEHB Charity |  |  |  |  |  |  |  |  |  |  |  |  |  |  |  |  |  |  |  |
| 1. Prepare material for dissemination |  |  |  |  |  |  |  |  |  |  |  |  |  |  |  |  |  |  |  |
| 1. Study Management Group meetings |  |  |  |  |  |  |  |  |  |  |  |  |  |  |  |  |  |  |  |
| 1. Study Steering Group meetings |  |  |  |  |  |  |  |  |  |  |  |  |  |  |  |  |  |  |  |

1. **Research Governance**

UoB is the Study sponsor. The Study will be conducted in accordance with the principles of the Research Governance Framework for Health and Social Care, with due consideration of it being a feasibility and pilot study. R&D approval will be sought from QEHB and SRFT. The CI will take overall responsibility for the Study and a PI has been identified at each participating hospital.

- 1. **Data Management**

The Study will follow GCP and SOPs. All data will be collected using standardised proforma (including patient intervention data), and anonymised data stored securely at the UoB.

- 1. **Protocol violation or withdrawal of treatment**

Participants who receive a treatment other than that randomised will remain in the Study and their data will be included in analyses. An intervention will be withdrawn if it leads to an unacceptable number of serious or adverse events for individuals randomised to it.

- 1. **Study Management Group (SMG)**

The CI chairs a SMG comprising two users (who have recently undergone first-time spinal discectomy), two physiotherapists from QEHB (including the Principal Grant holder), two physiotherapists from SRFT, the Co-ordinator for SRFT and a research methodologist /statistician. The SMG will meet face-to-face or via phone-conferencing every three to four months and will monitor progress of the Study and address any management, ethical or academic issues.

- 1. **Study Steering Group (SSG)**

A combined Study Steering Group and Data Monitoring & Ethics Committee (SSG) will be established, in keeping with the Study size. The SSG will comprise a Chair (an experienced researcher), a statistician and a user, all independent of the Study; and, the CI (or representative), and two other members from the SMG. The SSG will meet face-to-face or via phone-conferencing (according to individual preference) every five to six months. Its remit will be to: a) review relevant information from the Study team to oversee progress of the Study towards achieving its objectives; b) consider any adverse events; c) protect the rights of Study participants.

Records will be kept of all meetings and stored in a locked filing cabinet at UoB.

- 1. **Serious and unexpected adverse events**

There are unlikely to be any serious side effects from the interventions. Potential side effects could be worsening of symptoms. Participants allocated to the 1-1 physiotherapy intervention and leaflet will be monitored by the treating physiotherapist, as per routine practice. Participants who receive no treatment additional to the patient information leaflet (which is the normal situation in many hospitals) will be given a named contact (at each site) and telephone number in case of incident.

All adverse events will be reported to the approving Research Ethics committee and to SSG for discussion. A serious adverse event will be defined as one that requires hospitalisation as a result of the intervention, or where treatment causes unwarranted distress to a participant.

1. **Ethical Considerations**

The Study will be approved by the West Midlands – Solihull Research Ethics Committee prior to commencement. Site specific issues for QEHB will be approved by the Research and Development Directorate, Queen Elizabeth Hospital, Birmingham. Site specific issues for SRFT will be approved by the Research and Development Directorate, Salford Royal NHS Foundation Trust, Salford.

**References**

Arain M et al. What is a pilot or feasibility study? A review of current practice and editorial policy. BMC Medical Research Methodology 2010; **10:**67doi:10.1186/1471-2288-10-67.

Barrios C, Ahmed M, Arrotegui J et al. Microsurgery versus standard removal of the herniated lumbar disc: A 3 year comparison in 150 cases. Acta Orthopaedica Scandanavia 1990; 61(5):399-403.

Beurskens AJHM, de Vet HCW, Koke AJA. Responsiveness of functional status in low back pain: a comparison of different instruments. Pain 1996; 65: 71-76.

Browne RH. On the use of a pilot sample for sample size determination. Statistics in Medicine 1995;14:1933–1940.

CBO. The Lumbosacral Radicular Syndrome, Dutch Institute for Healthcare Improvement. Centraal Begeleidings Orgaan Report 2008.

Craig P, Dieppe P, Macintyre S, Mitchie S, Nazareth I, Petticrew M. Developing and evaluating complex interventions: the new Medical Research Council guidance. BMJ 2008;337:a1655 doi: 10.1136/bmj.a1655.

Deyo R, Battie M, Beurskens A et al. Outcome measures for low back pain research. A proposal for standardised use. Spine 1998; 23:2000-2013.

Donceel P, Du Bois M. Predictors for work incapacity continuing after disc surgery. Scand J Work Health Environ 1998; 25:264–271.

Donceel P, Du Bois M. Predictors for work incapacity continuing after disc surgery. Scand J Work Health Environ 1999; 25:264-271.

Foster MR. Herniated disc prolapse. eMedicine.com 2007. Accessed on 14.11.08; available from <http://www.emedicine.com/orthoped/topic138.htm>

Hakkinen A, YlinenJ, Kautiainen H et al. Effects of home strength training and stretching program versus stretching alone after lumbar disk surgery. Archives of Physical Medicine and Rehabilitation 2005; 86:865-870.

Hertzog MA. Considerations in determining sample size for pilot studies. Research in Nursing & Health 2008; 31: 180-191.

HES online. Primary Diagnosis: 3 character tables. The Health and Social Care Information Centre. Accessed 20.1.10; available from http://www.hesonline.nhs.uk

ICH Topic E6(R1). Guideline for good clinical practice. Step 5. Note for guidance on good clinical practice. CPMP/ICH/135/95. EMEA 2006.

ICH Topic E9. Statistical Principles for clinical trials. Step 5. Note for guidance on statistical principles for clinical trials. CPMP/ICH/363/96. EMEA 2006.

Kitzinger J. Qualitative research: introducing focus groups. BMJ 1995; 311:299-302.

Kitzinger J. Chapter 3 Focus Groups. In: C. Pope & N. Mays (eds.) Qualitative research in health care. Pages 21-31. Blackwell Publishing. BMJ Books.

Lancaster GA, Dodd S, Williamson PR. Design and analysis of pilot studies: recommendations for good practice.J Eval Clin Pract 2004; 10:307-12.[

](http://www.biomedcentral.com/sfx_links.asp?ui=1471-2288-10-67&bibl=B3)

Linton S. A review of psychological risk factors in back and neck pain. Spine 2000; 25(9):1148-1156.

Maniadakis N, Gray A. The economic burden of low back pain in the UK. Pain 2000; 84:95-103.

MacPherson H. Pragmatic clinical trials. Complementary Therapies in Medicine 2004; 12:136-140

McGregor AH, Dicken B, Jamrozik K. National audit of post-operative management in spinal surgery, BMC Musculoskelet Disord 2006; 7: 47. Published online 2006 May 31. doi: 10.1186/1471-2474-7-47.

McGregor AH, Burton AK, Sell P et al. The development of an evidence-based patient booklet for patients undergoing lumbar discectomy and un-instrumented decompression. European Spine Journal 2007; 16(3):339-346.

Moher D, Hopewell S, Schulz KF, Mintoru V et al. Consort 2010 explanation and elaboration: Updated guidelines for reporting parallel group randomised trials. International Journal of Surgery. 2012. 10:28-55

MRC (Medical Research Council). Developing and evaluating complex interventions: new guidance. www.mrc.ac.uk/complexinterventionsguidance. 2008. Accessed 16/10/2008

Ong BN, Hooper H (2003). Involving users in low back pain research. Health Expectations 6(4):332-341.

Ostelo RWJG, Goossens MEJB, de Vet HCW et al. Economic evaluation of a behavioral-graded activity program compared to physical therapy for patients following lumbar disc surgery. Spine 2004; 29(6):615-622.

Ostelo RWJG, Costa LOP, Maher CG et al. Rehabilitation after lumbar disc surgery. Cochrane Database of Systematic Reviews 2008, Issue 4. Art no: cd003007. DOI: 10.1002/14651858.cd003007.pub2.

Ostelo RWJG, de Vet HCW. Clinically important outcomes in low back pain. Best Practice & Research Clinical Rheumatology 2005; 19(4):593-607

Raymond RWJG, Goossens MEJB, de Vet HCW et al. Economic evaluation of behavioural-graded activity program compared to physical therapy for patients following lumbar disc surgery. Spine 2004; 29(6):615-622.

Roland M, Torgerson DJ. Understanding controlled trials: What are pragmatic trials? BMJ 1998; 316:285.

Ross-McGill H, Hewison J, Hirst J, Dowswell T, Holt A, Brunskill P, Thornton JG. Antenatal home blood pressure monitoring: a pilot randomized controlled trial. British Journal of Obstetrics and Gynaecology 2000; 107:217–221.

Rushton A, Wright C, Goodwin P, Calvert M, Freemantle N. Physiotherapy rehabilitation post first lumbar discectomy: a systematic review and meta-analysis of randomised controlled trials. Spine 2011; 36(14): E961-72

Selkowitz DM, Kulig K, Poppert EM et al. The immediate and long term effects of exercise and patient education on physical, functional, and quality of life outcome measures after single level lumbar microdiscectomy: a randomized controlled trial protocol. BMC Musculoskeletal Disorders 2006; 7:70. DOI: 10.1186/1471-2474-7-70.

Shanyinde M, Pickering RM, Weatherall M. Questions asked and answered in pilot and feasibility randomized controlled trials. BMC Medical Research Methodology 2011; 11:117.

Staley K. Exploring Impact: Public involvement in NHS, public health and social care research. 2009; INVOLVE, Eastleigh.

Stephenson J, Imrie J. Why do we need randomised controlled trials to assess behavioural interventions? BMJ 1998; 316:611-613.

Treasure T, MacRae KD. Minimisation: the platinum standard for trials? Randomisation doesn't guarantee similarity of groups; minimisation does. BMJ 1998; 317:362-3.

Vlaeyen JWS, Kole-Snijders AMJ, Boeren RGB, van Eek H. Fear of movement/(re)injury in chronic low back pain and its relation to behavioral performance. Pain 1995; 62:363-72.

Williamson E, White L, Rushton A. A survey of post-operative management for patients following first time lumbar discectomy. European Spine Journal 2007; 16(6):795–802.

WHO. International Classification of Functioning, Disability and Health: ICF. Geneva, Switzerland: World Health Organisation, 2001.

WHO. WHO Technical Report Series. The burden of musculoskeletal conditions at the start of the new millennium. Geneva; WHO 2003
